# Smartphones, citizen science, and the fight against gender-based violence in rural Tanzania

**DOI:** 10.3389/fgwh.2025.1490918

**Published:** 2025-05-30

**Authors:** Chandler Klein, Patty Kostkova, Herry Kasunga, Janet Chapman

**Affiliations:** Department of Risk and Disaster Reduction, University College London, London, United Kingdom

**Keywords:** citizen science, gender-based violence, digital exclusion, ICT4D (ICT for development), female empowerment, low and middle income countries (LAMIC)

## Abstract

**Introduction:**

In rural regions where gender-based violence (GBV) is rampant and communities are largely offline and off-the-map, technology-enabled interventions are emerging to enhance women's quality of life. These initiatives offer opportunities to empirically test the efficacy of citizen science approaches to anti-GBV efforts and contribute to broader debates on the role of smartphones in women's empowerment. Despite the rapid growth of citizen science-driven GBV projects, rigorous evaluations of their impact remain scarce. At the same time, the presumed link between information communication technology (ICT) access and empowerment—as reflected in target 5.b of the UN Sustainable Development Goals (SDG)—remains contested, with empirical studies often suffering from methodological and conceptual shortcomings.

**Methods:**

We seek to fill this gap and produce insights relevant to community-based organizations (CBOs), governments, international bodies, and others tackling GBV and digital exclusion. We do this through a mixed-method approach, guided by contribution analysis (CA) as the mode of logical enquiry. We also apply a novel adaptation of Warshauer's framework of ICT access and Cattaneo and Chapman's model for empowerment to rigorously unpack the variables and the relationship between them. This work represents the first time these two conceptual models have been combined. It also serves as a rare example of a related empirical work offering high-resolution conceptual clarity. Specifically, it relies on primary survey and in-depth interview data collected from a range of project stakeholders in close collaboration with the two implementing CBOs.

**Results:**

The findings reflect positively on citizen science methodologies, demonstrating their cost-effectiveness, role in fostering informed communities, and ability to capture locally-grounded observations that would otherwise be out of reach. The results indicate a rise in GBV response interventions due to improved case identification using the approach. However, the link between smartphone access and personal empowerment is weak. Digital competency skills development was measurable but did not surpass a basic level. Smartphones were primarily used for entertainment and socializing rather than for improving life chances.

**Discussion:**

These findings challenge the assumption that digital access alone is a catalyst for empowerment. While being offline arguably begets marginalization, findings suggest the reverse is equally true: the marginalized have less chance to translate device ownership into meaningful access. Thus, we cannot rest on providing devices and training alone. Solutions must be holistic and take into account the social embeddedness of technology.

## 1 Introduction

GBV is defined by the United Nations as “harmful acts directed at an individual based on their gender” (1). It encompasses various forms of violence, including domestic violence, sexual harassment, rape, early marriage, and female genital cutting (FGC) and is recognized as both a human rights violation and a global health crisis (2). Globally, 31% of women aged 15–49 have experienced intimate partner violence (IPV) or non-partner sexual violence (NPSV), with Africa bearing the highest regional burden at 36%. Tanzania follows this trend, with 38% of women affected (3), emphasizing the urgency of addressing GBV in this context.

**Figure 1:**
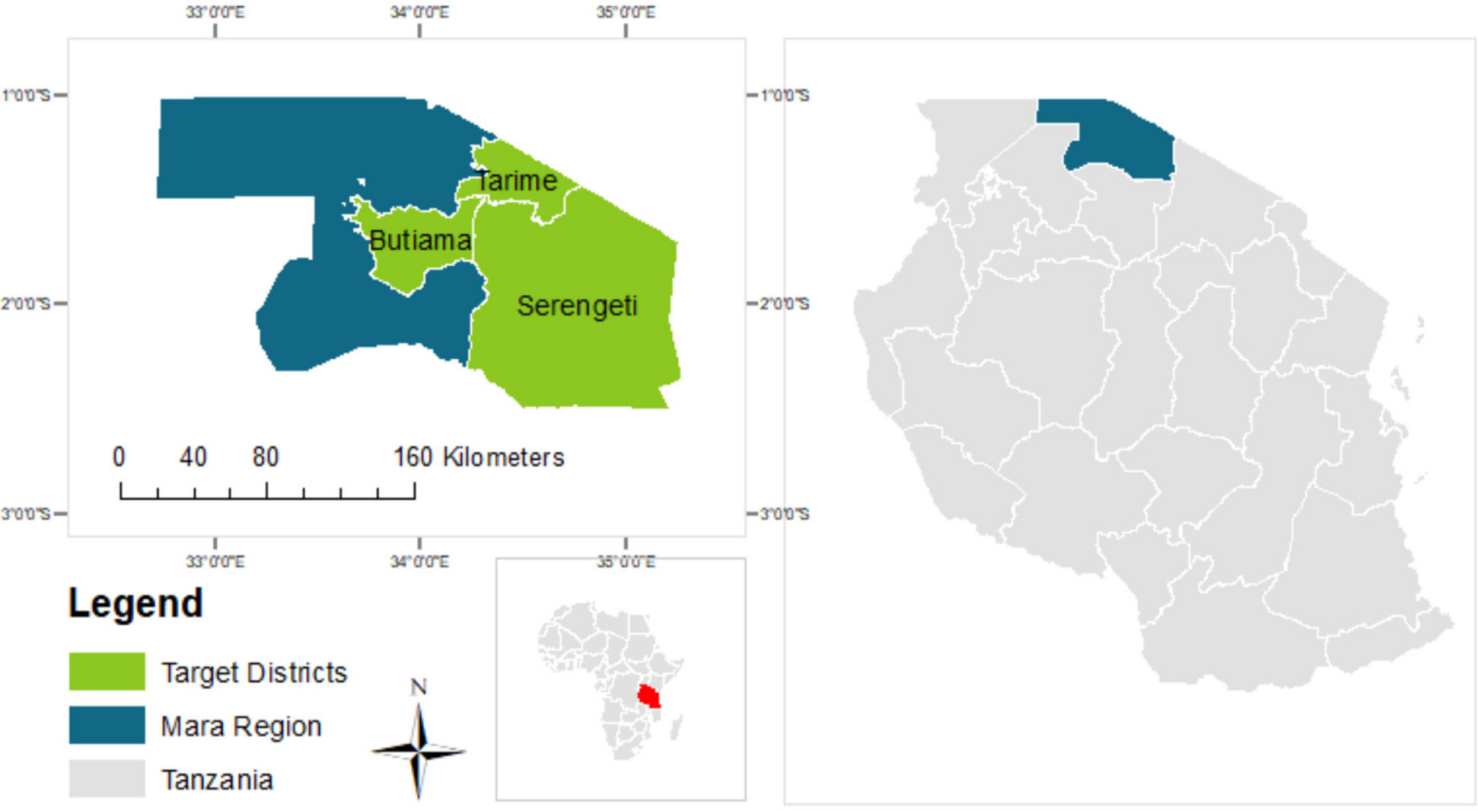
DC project area of implementation (original figure).

In parallel, digital exclusion remains a pressing challenge. As of 2021, approximately 2.9 billion people worldwide remain offline, with low-income and rural communities disproportionately affected (4). Sub-Saharan Africa has the world's lowest internet penetration, with only 33% of the population connected, compared to 90% in high-income countries (5). In Tanzania, only 25% of women use mobile internet, compared to 40% of men, reflecting a significant gender gap in digital access (6). With the internet dubbed the “electricity of the informational era”, some argue this puts such communities at a grave disadvantage, perpetuating dynamics of exclusion and disempowerment (7).

The Mara Region, a rural area of northern Tanzania, epitomizes these challenges. Within this context, there are strikingly high rates of GBV against girls and women, including domestic violence and female genital cutting (8). At the same time, access to smartphones and other digital ICT lags behind much of the world. While precise figures are unavailable, anecdotally, smartphones – the device type responsible for the bulk of web traffic - are very uncommon in these remote villages (9).

This Digital Champions (DCs) Project interlink these two seemingly disparate issues through a single solution: equipping women volunteers, known as Digital Champions (DCs), with smartphones and digital training. The intervention focuses on three districts within the Mara Region—Serengeti, Butiama, and Tarime—which are among the seven administrative districts of the region and are illustrated in Figure 1 below. Using the Kobo Collect mobile application, DCs identify and report GBV cases in their communities. Additionally, they contribute missing geographic data to OpenStreetMap via Maps.Me, aiding organizations such as Hope for Girls and Women (Hope), the Association for the Termination of Female Genital Mutilation (ATFGM), the Gender Desk Police (GDP), and the Social Welfare Office (SWO) in reaching and assisting victims in hard-to-access areas. This falls under the umbrella of citizen science approaches, which involves public participation in the production of data. Further, the project intends that the device and training function as a launching pad from which DCs continue to expand their phone use and become empowered to take actions for the betterment of themselves and society.

This impact evaluation assessed to what extent project activities improved GBV interventions through a citizen science approach and empowered DCs through smartphone access. The answer promises a tangible impact to project implementers. Moreover, it contributes empirical insight into the merits and demerits of citizen science approaches to GBV prevention and response efforts. Further, it weighs in on the highly contested debate of the transformative power of ICTs, through a specific focus on Tanzania at the intersection of rurality and gender.

## 2 Literature review

### 2.1 Digital ICT-enabled citizen science approaches to GBV prevention and response

Citizen science, broadly defined as the production of scientific knowledge through public participation, has a long-standing role in public health (10). More recently, citizen science methodologies—such as community-led data collection, crowdsourced reporting, and participatory mapping—have gained recognition as tools for GBV prevention and response. Digital platforms have expanded the reach of these approaches, enabling large-scale reporting, geospatial analysis, and real-time data visualization. For example, several projects have cropped up which make use of the ubiquity of smartphones with GIS capabilities to spatially track and publish incidents of sexual harassment (11–13). The Centre for Life Change Project teaches community members in Uganda to map safe spaces and GBV services while the Our Voice Project mobilizes students on college campuses to digitally collect data about sexual violence and develop solutions (14, 15).

This growth in digital citizen science aligns with broader trends in women's health, where mobile interventions are increasingly used to enhance knowledge and access to care. Projects like MANTRA in Nepal have leveraged serious games to improve maternal and neonatal health literacy, particularly in low-literacy communities (16). Similarly, the Neotree platform has demonstrated the potential of digital decision-support tools for newborn care in low-resource settings (17). However, while engagement of women with technology in Nepal has demonstrated a step forward, gender sensitive interventions in low- and middle-income countries are still challenge to local societal practice and patriarchal orders (18).

This collection of digital ICT-enabled citizen science initiatives is lauded for their cost-effectiveness, sustainability, and effectiveness (13, 14, 19). The logic follows that involving communities is essential to gleaning locally-grounded insights, can stimulate critical reflection over GBV issues, and foster a sense of ownership over solutions (20, 21). However, concerns persist regarding data reliability, ethical risks, and the potential for misinformation (22). Challenges such as inconsistent data verification, underreporting due to stigma, and safety risks for participants highlight the need for robust methodologies and ethical safeguards. While other public health fields have demonstrated that well-structured citizen science projects can yield high-quality data (23), evaluations of GBV-related initiatives remain scarce.

### 2.2 ICT access and empowerment

#### 2.2.1 What is ICT access?

ICTs encompass a heterogeneous set of technological tools and services such as phones and computers, used to produce, share, manage, and transform information (24). While this is agreed, understanding of what is meant by ICT access varies. On the one hand, those embracing the traditionalist perspective equate it to meaning physical access to technological infrastructure combined with relevant technical competencies. The opposing view, which is grounded in the tradition of constructivism, considers technology to be embedded within the contexts in which they operate, mediating how they are viewed and used. Warshauer (7) presents a useful framework for conceptualizing this more expanded view of ICT access, which he argues relies on resources positioned against four overarching axes summarized in Table 1 below. These resources have been deconstructed similarly by other researchers (25–27).

**Table 1:** Warshauer (7) resources that contribute to ICT access.

Physical resources	Digital resources	Human resources	Social resources
Access to devices and telecommunication connections	Digital online material online (is it relevant to people's needs? In the appropriate language?)	Education and literacy, that is relevant for ICT use	Community, institutional, and societal structures that support

#### 2.2.2 What is empowerment?

Scholars and practitioners generally agree that fundamentally, empowerment it is about choice and power. Nonetheless, there is wide variation and imprecision in how it is defined. For instance, Kabeer (28) defines the term as “the process by which those who have been denied the ability to make strategic life choices acquire such an ability” (28). Alsop and Heinsohn (29) adds to this definition “the capacity to transform choices into desired actions and outcomes”, while Mechanic (30) considers it to be “a process in which individuals learn to see a closer correspondence between their goals and a sense of how to achieve them, and a relationship between their efforts and life outcomes” (29, 30).

Selected models and frameworks which attempt to deconstruct empowerment into measurable components are presented in Table 2. Cattaneo and Champan's (32) model, which informs the approach applied by the present study, offers a number of advantages. Firstly, it best captures the oft-cited logic of empowerment as a dynamic process. Moreover, it acknowledges empowerment as a bottom-up process by explicitly naming personally-defined goals as animating the process. Further, it gives attention to how context mediates the process and concepts are amenable to measurement. The primary weakness of this model is its narrow view of competence as skills, which ignores social and material assets as being influential.

**Table 2:** Selected models and frameworks of empowerment.

Model/framework	Key tenants
Nomological network of psychological empowerment (31)	*1. Intrapersonal Component* – How people view themselves. *2. Interactional Component* – Understanding of context and behavioural options. *3. Behavioural component* – Actions taken with a view to achieving outcomes.
Empowerment conceptualization (28)	*1. Resources –* Refers to material, human, and social resources *2. Agency –* Decision making processes *3. Achievements –* Wellbeing outcomes
Model for empowerment (32)	*1. Personally meaningful and power-oriented goals* *2. Self-efficacy –* Individuals perception about his/her ability *3. Knowledge* – Understanding of routes to achieving goals *4. Competence* – Skills to execute the needed actions to accomplish goals. *5. Action* – Acts in service of goal achievement *6. Impact* – The results of the actions in reference to the goal. ***There are dynamic relationships between all of the above-mentioned elements. In particular, the model emphasizes the role of social context

#### 2.2.3 How can ICT enable empowerment?

The belief that ICT access empowers individuals has gained widespread prominence in the academic literature and development policies (33). For example, this presumption is embraced in target 5.b of the UN Sustainable Development Goals (SDG) (34). Some scholars cast doubt, arguing that ICT interventions individualize solutions to problems that are rooted in structural inequities (35, 36). Many empirical studies have concluded that ICTs precipitate changes in self-confidence and self-efficacy. However, evidence of tangible life improvements has been more muted, contributing to the belief that ICT optimism is naïve (37–39).

Within the empirical works, ICT access is often equated to physical access to technological infrastructure combined with relevant technical competencies and neglects the role of enveloping social structures and other factors in mediating use (40, 41). Further, empowerment is often imprecisely defined and not grounded in relevant conceptual models (37, 38, 42, 43). With most studies, it is difficult to establish the causal relationship in the absence of longitudinal data or counterfactuals. Further, there is a crop of works that exclusively relies on surveys (40, 44). This requires a pre-definition of intended empowerment outcomes, which is at odds with notion of self-determination, the very essence of empowerment.

### 2.3 Gaps in the literature

There are three overarching observations of the existing literature worth reiterating. First, there is limited empirical literature on the efficacy of digital ICT-enabled citizen science approaches to GBV prevention and response. Secondly, the optimism surrounding the potential of ICT as a tool for empowerment is highly contested. This unresolved debate extends to smartphones use among women in resource-constrained settings, which to date has received limited attention. Thirdly, many empirical works considering the relationship between ICT and empowerment are atheoretical, have poorly defined causal mechanisms, and rely on reductive quantitative methodologies with weak internal validity. The present study attempts to build on the current state of knowledge and overcome identified gaps.

## 3 Methodology

### 3.1 Design overview

This impact evaluation study followed a mixed-methods contribution analysis (CA) approach, which is used widely in evaluation circles (45). The defining strength of CA is that it offers a systematic way of reducing uncertainty about causality between the project activities and the desired changes it seeks to effect in the absence of a counterfactual. This logic of enquiry is executed using a mixed-methods cross-sectional design. Specifically, it is guided by the research questions posed in Table 3 below.

**Table 3:** Study research questions.

#	Research questions
1	To what extent did the project activities contribute towards increasing timely and appropriate GBV interventions through a smartphone-enabled citizen science approach? What factors contributed to or hindered achievement of this outcome?
2	To what extent did the project activities contribute to empowering DCs to act as positive change agents both within their own lives and within the community? What factors contributed to or hindered achievement of this goal?

### 3.2 Theory of change (ToC) and conceptual framework development

**Figure 2:**
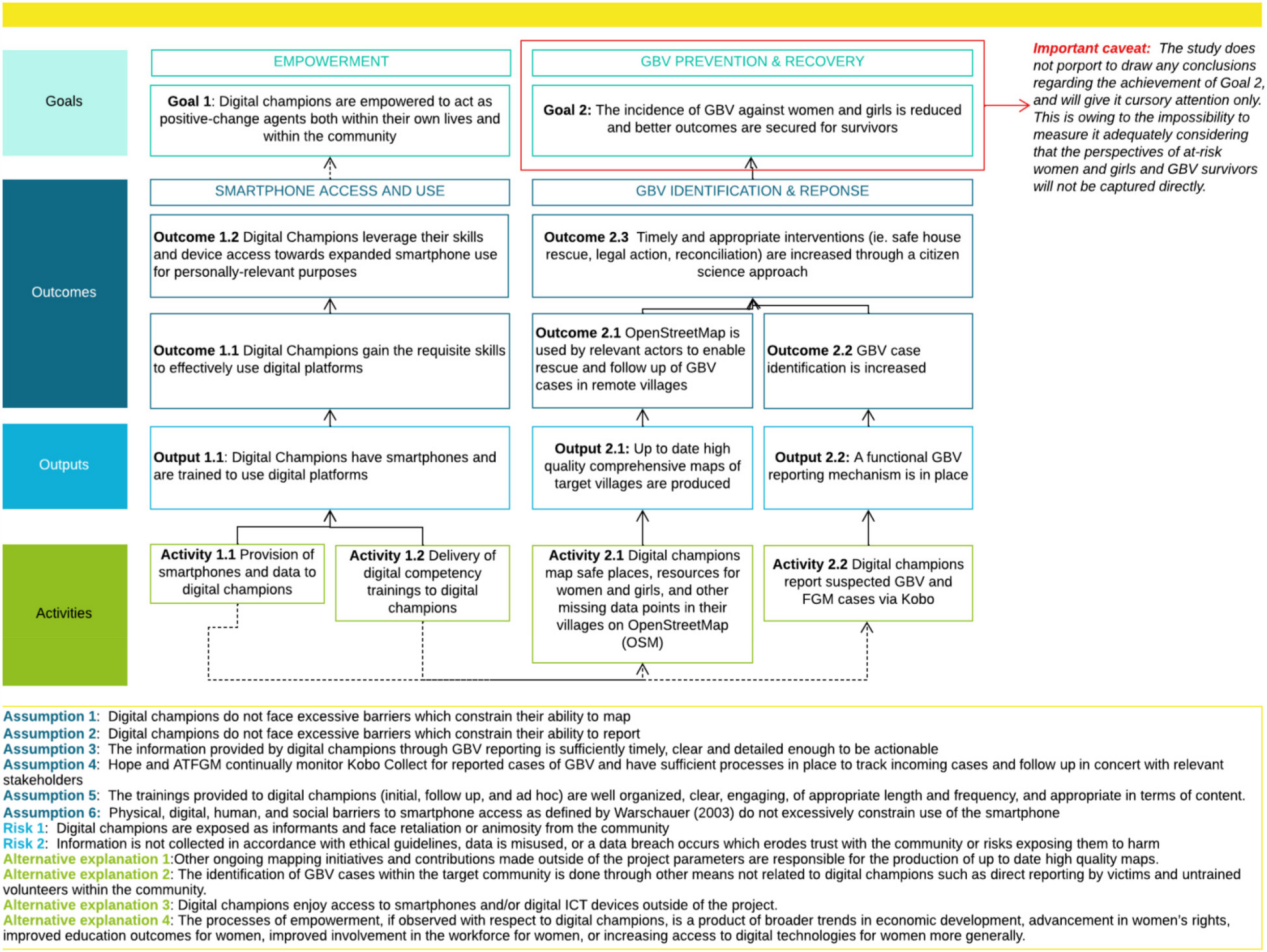
DC project postulated ToC (original figure).

Developing a postulated ToC (Figure 2) was a key step in the CA approach. A ToC is a logical model which articulates the causal linkages between the collection of project activities to the successive levels of desired outcomes and changes it seeks to create. Included in the ToC are the assumptions to hold, risks to avoid, and alternative explanations to eliminate for the integrity of the ToC to remain intact. It provided the framework against which evidence was collected and tested.

**Figure 3:**
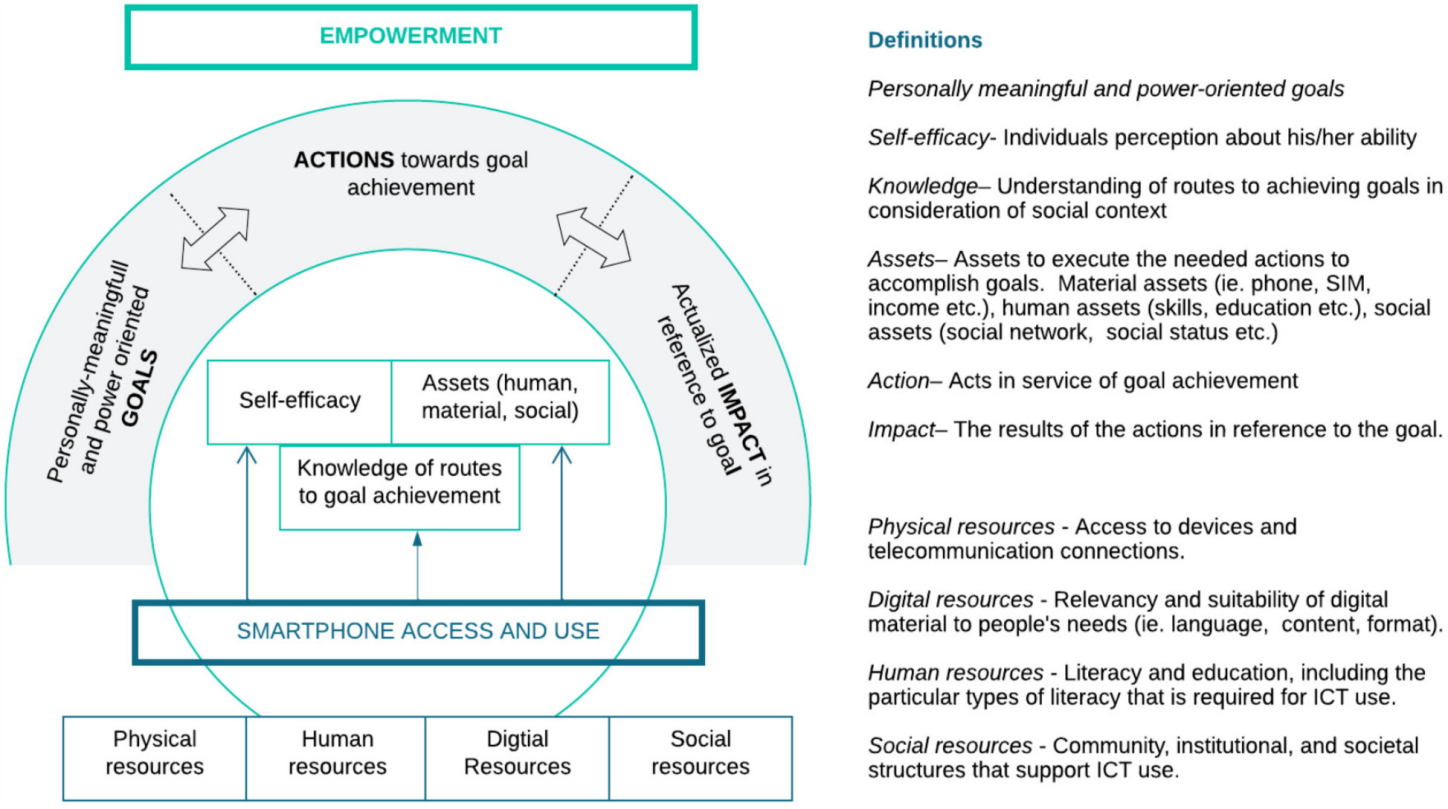
Model of ICT access and empowerment adapted from warshauer (7) and cattaneo and champan (32) (original figure).

Further, to disentangle the link between outcome 1.2 on smartphone access and goal 1 on empowerment, the model shown in Figure 3 was developed. This model draws on Warshauer's framework of ICT access and on Cattaneo and Chapman's model of empowerment. Warshauer (7) argues that ICT access extends beyond the presence of a device, and rather relies on resources positioned against four overarching axes, physical, digital, human, and social. Cattaneo and Champan's (32) model, was selected because it captures the logic of empowerment as a dynamic process, acknowledges empowerment as a bottom-up process, gives attention to how context mediates the process, and includes concepts amenable to measurement. The variable of competence within Cattaneo and Chapman's original model was replaced with a variable called assets, inspired from Kabeer's (28) framework of empowerment. This adaptation allowed for a more expanded interpretation of the types of assets beyond skills which an individual may require to set actions into motion.

### 3.3 Data collection methods, sampling, and data analysis techniques

A mixed method approach was applied to combine primary survey and in-depth interview data collected from a range of project stakeholders in close collaboration with the two implementing CBOs.

#### 3.3.1 Survey with DC respondents and statistical analysis

The survey was conducted over the phone with a simple random sample of 117 DCs, of which 86 were women, in order to achieve results generalizable to a 95% confidence interval and 10% margin of error (MoE). A descriptive analysis was conducted. Further, in order to assess the statistical significance of the change in digital skills competency, a Wilcoxon matched-pairs signed-rank test was used, which is a non-parametric method to compare before and after across the same data subjects.

#### 3.3.2 In-depth interviews with DCs and project stakeholders

Thematic analysis was applied to interviews conducted with four categories of project stakeholders, including eight DCs, five Hope and ATFGM staff, two GDP, and two SWO. Half of the DCs were purposefully sampled to best exemplify the changes the project seeks to create while the remaining half were those that encountered challenges per survey results, as a means investigate the enablers and barriers to change. The remaining stakeholders were purposefully selected according to level of interaction with the project. The number of interviews was set according to time and resource constraints. Data collection for both the survey and interviews took place between May 29th and August 23rd 2022. Transcripts were coded to identify recurring themes in relation to the key elements identified in the theory of change and conceptual framework.

## 4 Results

The results are organized under the headers of the two overarching research questions. Consistent with a CA approach, findings are presented in reference to the outputs, outcomes, goals, risks, and assumptions that comprise the postulated ToC.

### 4.1 Increasing GBV interventions through a digital smartphone-enabled citizen science approach

#### 4.1.1 Output 2.1: Up to date high quality comprehensive maps of target villages are produced

Survey data indicates that mapping contributions made by DCs have been relatively modest since the training held a year or more prior, with 67% of surveyed DCs reporting some level of mapping activity (Table 4).

**Table 4:** Cumulative # of hours spent mapping by respondent and demographic (%).

Demographic	*n*	0 h	<1 h	1–3 h	3–10 h	10+ h
All	117	33%	28%	26%	8%	5%
Women	86	37%	30%	21%	6%	6%

#### 4.1.2 Assumption 1: DCs must not face excessive barriers which constrain their ability to map

When prompted on the top mapping challenges, a lack of data and no signal were the most cited reasons (Table 5). ATFGM and Hope have irregularly supplied credit and many DCs are not willing to top up at their own expense. “When we get money, we try to give them”, explained a staff member. Moreover, poor signal, which is particularly commonplace in Serengeti, is a major inconvenience but largely unavoidable owing to Vodafone's coverage monopoly. Twenty-four percent cited skills as a barrier to map, something which particularly affected women. One such DC from Serengeti noted that, “The application is difficult to understand, and I could not see how to add points to the map”.

**Table 5:** Challenges faced by surveyed DCs when mapping by respondent and demographic (%).

Challenge	Women	Total
***n***	**86**	**117**
Limited data	45%	44%
No signal	30%	31%
Lack knowledge	29%	24%
No challenge	9%	12%
Already mapped	9%	12%
Phone lost or broken	9%	8%
No time	8%	7%
Phone not able to be easily charged	8%	6%
Other	3%	4%

#### 4.1.3 Outcome 2.1 OpenStreetMap is used by relevant actors to enable rescue and follow up of GBV cases in remote villages

Hope staff, ATFGM staff, the GDP, and SWO have unequivocally stated that OpenStreetMap is used frequently and has been instrumental in improving ease of rescues and GBV case follow up. One explained that “The maps help us to plan to go to the event, how many kilometres it will take, how much fuel is needed”. Another staff added that “It has brought a very big change” while a third noted that “If you can see there is a church or police station in Matari, you can say hey, maybe take the victim there … so you can see the importance of labelling structures in making decisions”.

#### 4.1.4 Output 2.2: A functional GBV reporting and response mechanism is in place

DCs are reporting according to their own self-reporting and GBV report extractions from Kobo (Table 6). Beyond physical obstacles to reporting (Table 7), interview data suggests that a poor understanding of GBV concepts can hinder case identification. Further, results indicate that 35% of surveyed male DCs compared to only 16% of female DCs consider the difficulty in identifying GBV cases as a key impediment. Interview data suggests this is linked to the way information on abuse is circulated more freely within female circles, with one DC explaining, “It is part of our culture. Normally what is happening inside the house people share outside…especially with other women”.

**Table 6:** Cumulative # cases reported on kobo per DC by demographic (%).

Demographic	*n*	0 cases	1–5	6–20	21–50
All	117	36%	45%	15%	4%
Women	86	36%	48%	14%	2%

**Table 7:** Challenges faced by DCs when reporting by respondent and demographic (%).

Challenge	Women	Total
***n***	**86**	**117**
Limited data	44%	46%
No signal	33%	29%
Difficulty to identify cases	16%	21%
No challenges	17%	18%
Phone lost or broken	13%	11%
Lack knowledge	14%	10%
Other	6%	5%
Fear community backlash	5%	4%
No time	3%	3%
Difficulty charging phone	5%	3%

DCs were limited to select the top three challenges they encountered.

Others speculated that the failure of ATFGM and Hope to consistently acknowledge and respond to DC GBV reports leaves some DCs unmotivated to report. This is corroborated by some accounts of DCs, “You don't know if the organization received the report even or did anything. This makes you feel like they are not paying attention and like everything is pointless”. One SWO added that “My number one suggestion is for the project to better address how to share information back to DCs. They will give up if they raise many cases but they are not resolved”.

#### 4.1.5 Risk 1: DCs are exposed as informants and face retaliation or animosity from the community

While all stakeholders acknowledged the risk that DCs could be exposed as informants, the perception of the likelihood of that risk is relatively low. Only 4% of DCs cited fear of community backlash (Table 7), although several of those interviewed shared that they were under suspicion.

#### 4.1.6 Assumption 3: The information provided by DCs through GBV reporting is sufficiently timely, clear, and detailed enough to be actionable

For a functional GBV reporting mechanism (output 2.2), information reported must be usable. ATFGM staff, Hope Staff, SWO and GDP concede that there are limitations in data quality. Examples of issues include missing information, contradictory information, and mischaracterization of the GBV cases. However, a complete and accurate picture typically is assembled when a follow up on the ground is made so this does not significantly interfere with the process, according to relevant actors.

Several have doubted DC's mastery of GBV topics. Of the 98% of DCs who agreed that they require more training, GBV emerged as topic most in demand. As put by one GDP, “DCs need more knowledge apart from reporting, to understand GBV in detail and its types”. This is underscored by an anecdote shared by a SWO, who asserted that “If the DC had a better grasp of child protection principles, this situation [sexual abuse of a child] could have been avoided”.

#### 4.1.7 Assumption 4: Hope and ATFGM continually monitor kobo collect for reported cases of GBV and have sufficient processes in place to track incoming cases and follow up in concert with relevant stakeholders

Collectively, staff from both organizations are confident that all submitted cases are checked on a daily or weekly basis according to the time of year. At the same time, interview data highlights weaknesses in their information management (IM) system such as a lack of an automated notification system or integration with the case management system. Additionally, case data is often transmitted via phone calls due to connectivity issues, fragmenting storage locations and hindering case tracking.

#### 4.1.8 Outcome 2.2 GBV case identification is increased

All evidence is supportive that DC reporting has translated into a dramatic increase in the number of GBV cases identified. One SWO noted that “I would estimate 80% of the cases I see are from DCs and the remaining 20% are raised by the village leaders”. This observation is further supported by the Butiama Office statistics which shows how the cases tripled from 2018/2019 to 2020/2021 concurrently with the introduction of the DCs. One stakeholder noted that “They act like our eyes because we are not able to look around every village. It saves money and time”.

#### 4.1.9 Outcome 2.3 timely and appropriate interventions (i.e., safe house rescue, legal action, reconciliation) are increased

For the purposes of the study, we were not able to access internal records that would shed light on how many reports from DCs precipitate an intervention. Anecdotally it seems that this equated to a higher number of GBV interventions. However, as one staff member explained, “The budget it is not enough to clear out the cases the DCs report”.

### 4.2 Empowering DCs through smartphone access

#### 4.2.1 Outcome 1.1 DCs gain the requisite skills to effectively use digital platforms

Findings suggest that most DCs achieved some level of basic digital competency from the loan of smartphones and training provided, with the mean retrospective pre-test digital skills competency score at 6.3 points compared to the mean post-test score of 14.0 out of a total possible of 16 points. A Wilcoxon matched-pairs signed-rank test indicates a statistically significantly improvement in scores (*p*-value < 0.5) for all skills domains (Table 8).

**Table 8:** Wilcoxon matched-pairs signed-rank test analysis of pre and post scores by digital skill (*n* = 117).

Digital skill	Mean pre score	Mean post score	Mean difference	*p*-value
Turn phone on/off	1.31	2.00	0.65	3.728 × 10^−10^*
Add credit	1.18	2.00	0.77	1.03 × 10^−11^*
Install applications	1.05	1.84	0.74	4.2 × 10^−11^*
Email	0.83	1.28	0.42	4.831 × 10^−07^*
Internet search	0.89	1.52	0.59	2.76 × 10^−09^*
Social media	0.96	1.85	0.84	1.98 × 10^−12^*
KOBO	0.01	1.83	1.71	<2.2 × 10^−16^*
Maps.Me	0.01	1.63	1.53	<2.2 × 10^−16^*

* Indicates significance (*p* < 0.05).

#### 4.2.2 Outcome 1.2 DCs leverage their skills and device access towards expanded smartphone use for personally-relevant purposes

According to the survey results presented in Table 9, 65% of DCs reported that they always used the phone for personal reasons. As highlighted in Table 10, the most frequently cited use is to call and text, take pictures, and use applications such as Youtube, Whatsapp, Instagram, Twitter and Facebook. Only 23% reported using the internet search browsers. When prompted on this, most DCs appeared to have minimal familiarity with this function. Interviews with DCs suggest that phone use is primarily oriented around entertainment and social purposes, with very limited examples of phone use for employment, business civic engagement, education, accessing news etc.

**Table 9:** Frequency of smartphone use for personal reasons by respondent (%).

Demographic	*n*	Always	Sometimes	Never	Total
All	117	65%	24%	11%	100%
Women	86	64%	23%	13%	100%

**Table 10:** Uses of smartphones for personal reasons beyond project (by %).

Uses	Women	Total
***n***	**75**	**104**
Call and text	99%	99%
Take pictures	60%	57%
Applications	45%	45%
View preloaded materials	49%	43%
Listen to music	40%	38%
Internet browser	21%	23%
Online marketplace	3%	3%
Content creation	4%	3%
Play games	3%	3%

#### 4.2.3 Assumption 6: Physical, digital, human, and social barriers to smartphone access as defined by Warschauer (7) do not excessively constrain use of the smartphone

Per the survey results, physical resource challenges were a significant hinderance. As highlighted in Table 11, this includes previously detailed challenges. One DC in Butiama explained, “When Hope was supporting us with data I would browse the internet for farming information, but now it's a challenge so it has been a while”. In reference to the phone, interview data reveals there are issues related to storage capacity, batterie life, non-functioning chargers. Further, while DCs themselves do not perceive a human resource deficit, qualitative data suggests that skills beyond a basic level are limited. When prompted on the difference between different types of content on the mobile internet, such as advertisements, public comments, and news articles, the level of understanding was rudimentary. Further, knowledge on how to discover new and useful services, applications, and information as well as create content beyond social media platforms, such as in an online marketplace, was non-existent for most respondents. In reference to social resources, DCs' acknowledge that beyond the information shared by Hope and ATFGM, they relied on their social circle as a resource on phone use. However, qualitative data suggests that this learning channel is limited. As one DC from Butiama noted, “I think others in my village normally use their smartphone for fun reasons only and not many people own one. I am not sure if they know what the internet is either”.

**Table 11:** % Of DCs who cite various resource challenges to extra-project phone use*.

Challenge	Category	Women	Total
***n***		**31**	**41**
Phone is lost or broken	Physical	23%	20%
Weak mobile signal strength	Physical	19%	15%
Not able to charge phone (lack electricity)	Physical	19%	15%
I don't have enough data	Physical	19%	15%
I don't know how to use other applications/ways to use the phone	Human	6%	5%
My family/community would not like it	Social	3%	2%
It is not encouraged by Hope/ATFGM	Social	0%	2%
I don't have time	Social	0%	0%
Things I care about (i.e., local news, crop price information etc.) are not available online or in a language in which I understand	Digital	0%	0%

* DCs were limited to select the top three challenges they encountered.

#### 4.2.4 Goal 1: DCs are empowered to act as positive-change agents both within their own lives and within the community

Per the empowerment model (Figure 3), the majority of DCs were not clearly able to articulate their goals. The qualitative evidence suggests that smartphones and participation in the project improved DCs self-efficacy, or perception about his/her own abilities. However, the link between smartphone access and improved knowledge of routes to goal achievement and improved assets in service of a personally-meaningfully goal are tenuous, with no solid examples provided. Moreover, from the collected data it is not possible to infer that any of these collective improvements have spurred action or precipitated transformative changes on the individual level in the social, economic, or political realm.

## 5 Discussion

### 5.1 Connecting the dots: linking project activities to increased and improved GBV interventions

This section focuses on research question 1, which asks to what extent project activities contributed towards increasing and enabling GBV interventions through a citizen science approach and what factors contributed to or hindered achievement of this outcome. The results in relation to this contribution narrative are strong as they relate to the causal chain involving GBV reporting but inconclusive in relation to mapping.

The evidence suggests that mapping contributions made by DCs have been relatively modest but taking place for the majority. Key barriers include network issues, insufficient data, and limited mapping confidence, highlighting areas for improvement. However, it is not possible to independently quantify or differentiate their contributions on OpenStreetMap from other users. Although actors confirm that they use the maps frequently and that they have substantially improved the speed and ease of their rescue efforts, uncertainty in earlier causal links limits confidence in attributing these outcomes to DCs' mapping efforts.

On the other hand, the evidence is largely consistent with the GBV reporting contribution story. Findings suggest that the DC-led GBV reporting mechanism is functional but has areas for improvement. While many DCs actively report cases, over a third face barriers such as network issues, limited data, poor phone quality, and gaps in GBV comprehension, which impact reporting consistency. Despite these challenges, staff and local authorities agree that DC reports are generally reliable and actionable. Findings suggest that DC reporting has significantly increased GBV case identification, with no alternative explanations for the rise. However, while increased reporting has led to more interventions, resource constraints and gaps in institutional coordination limit systematic responses.

The DCs Project showcases the potential of citizen science in GBV prevention and response, while underscoring the need for expert oversight, ethical safeguards, and institutional support to ensure data accuracy, participant safety, and sustainability. Stakeholders affirm that DC-led reporting significantly increased GBV case identification with minimal additional costs, generating otherwise inaccessible data. This reinforces the literature's assertion that citizen science is cost-effective and produces locally grounded insights, making it an approach worth considering for others implementing community-driven GBV interventions.

However, findings also align with broader concerns about data reliability in citizen science. While DC reports were generally actionable, inconsistencies in case classification and incomplete submissions underscore the need for enhanced training on GBV and reporting protocols. Encouragingly, all reports undergo expert review before action is taken, ensuring credibility. These lessons hold value for practitioners seeking to integrate citizen-generated data into formal response systems, highlighting the importance of balancing community engagement with expert validation.

Citizen science fosters community ownership, and the study suggests that DCs are deeply invested in addressing GBV, demonstrating commitment and pride in their role despite challenges. However, sustaining engagement requires visible impact. The study found that lack of feedback discouraged DC participation, highlighting the need for structured two-way communication and acknowledgment of reports—a consideration for any participatory initiative aiming for long-term engagement. While it is encouraging that the risk of being labeled a “whistleblower” did not strongly deter DC participation, the evidence suggests a critical need to strengthen safety measures for both DCs and survivors. Ensuring do-no-harm principles are embedded into reporting mechanisms, alongside training on anonymity and ethical reporting is essential.

### 5.2 Connecting the dots: linking project activities to the empowerment of DCs to act as positive change agents

This section seeks to shed light on research question 2: to what extent did smartphone access contribute to empowering DCs to act as positive change agents, and what factors contributed to or hindered the achievement of this goal? The results in relation to this causal narrative are ultimately weak. Nonetheless, it can be said with a reasonable degree of confidence that important strides were made in contributing to observed changes at the outcome level.

In line with the project's Theory of Change (ToC), all accounts confirm that training was delivered, and smartphones were loaned to DCs. Findings suggest that most DCs achieved some degree of basic digital competency, with statistically significant improvement in digital skills from the retrospective pre-test to post-test, indicating that these changes would likely not have occurred without the project. However, skill development plateaued at a basic level, with many DCs struggling to use more advanced smartphone features. This limitation was shaped by the narrow scope of training and the lack of exposure to digital tools within DCs' social networks. Research in digital inclusion similarly highlights that ICT training programs must extend beyond foundational skills, integrating digital literacy within locally relevant priorities to foster deeper engagement and long-term use (46). A key recommendation is to broaden training coverage to include mobile internet applications most relevant to digital champions' personally meaningful power-oriented goals (i.e., online marketplaces).

The project also contributed to increased smartphone use beyond project activities, though primarily for entertainment and social purposes. Given that many DCs had never used a smartphone before, the frequent and varied usage observed at the time of data collection represents a shift from the pre-intervention status quo. However, limited access to mobile data, poor phone quality, and inconsistent network connectivity were key constraints that restricted usage beyond basic applications. These findings reinforce the importance of ensuring that devices are of sufficient quality and that DCs receive regular data top-ups, particularly in the initial phases of engagement, to encourage more meaningful and sustained use of digital tools. Despite the potential of smartphones to expand opportunities in education, employment, and civic engagement, DCs largely engaged with a narrow range of applications and lacked awareness of the broader functionalities of the mobile internet, including tools such as Google search. This limited engagement reflects a well-documented challenge in ICT for development (ICT4D) programs, where access alone does not translate into transformative use unless paired with strategic training and resource support (47).

The data provides little evidence that project activities catalyzed an empowerment process aligned with the study's conceptual model. It is plausible to deduce from the evidence that smartphones and participation in the project improved DCs self-efficacy, or perception about his/her own abilities. This has been consistently highlighted by DCs. However, the link between smartphone access and improved knowledge of routes to goal achievement and improved assets in service of a personally-meaningfully goal are tenuous. Moreover, from the collected data it is not possible to infer that any of these collective improvements have spurred action, which per our conceptualization is a required step in the empowerment process. This aligns with the findings of much of the existing empirical work from the wider literature, which struggles to validate the presumption that ICT access precipitates transformative changes on the individual level in the social, economic, or political realm. Conversely, it offers support for the those that argue that empowering potential of ICT is overstated and overly optimistic. This highlights way in which ICT interplays with a complex web of overlapping social structures, suggesting that there are greater forces at work and devices and training alone may not be enough to set individuals on a path to making meaningful life improvements.

### 5.3 Limitations of the research and future direction

Inevitably, there are limitations to consider when reflecting on the discussion. Some variable operationalization relied on self-reported measures and thus carries a bias. For example, digital skills competency would be better measured through the direct testing of competencies. Additionally, the quality of the data may have been compromised by language barriers and the necessity of remote data collection. Additionally, while efforts were made to correct for uncertainty around causality, the methodology would be strengthened through longitudinal data collection. Additionally, to avoid doing harm, the perspective of GBV survivors and at-risk women and girls was absent from the study, which represents a fruitful area of enquiry for future evaluations.

## 6 Conclusion

This research uses the DC project as a case study to empirically test the largely unexplored question of the efficacy of citizen science for anti-GBV efforts and to weigh in on wider debates surrounding the transformative power of ICT. By focusing on a smartphone-based intervention targeting women in rural Tanzania, this study provides a unique empirical contribution in a context where such interventions remain largely unexplored. The findings are of direct relevance to actors globally who are investing in tackling challenges around GBV and digital exclusion.

A core contribution of this study is its methodological innovation. It applies a novel adaptation of Warshauer's framework of ICT access and Cattaneo and Chapman's model for empowerment, offering high-resolution conceptual clarity in assessing the relationship between technology access and empowerment. Unlike prior studies that reductively equate ICT access to device ownership, this study accounts for social structures, digital skills, and contextual barriers that mediate smartphone use. Moreover, the use of CA addresses internal validity shortcomings found in many ICT and empowerment studies, ensuring a more rigorous unpacking of causal relationships. This methodological framework is highly replicable for future research assessing ICT-driven development interventions.

Findings suggest that citizen science can be a valuable tool for GBV prevention response, particularly in enhancing case identification through community-led reporting. Specifically, it proved to be a cost-effective means of generating locally grounded insights, enabling authorities to access otherwise inaccessible information on GBV incidents. At the same time, it drew attention to the need for expert oversight to mitigate potential challenges with data reliability as well as the importance of do-no-harm measures to preserve participant safety. While the project effectively fostered community ownership in tackling GBV, the lack of feedback loops after reports were shared diminished motivation and could weaken long-term engagement. Nonetheless, GBV reporting by DCs translated into a dramatic increase in the number of cases identified. This in turn gave responders an increased opportunity to take action, albeit moderated by human and financial constraints.

On the question of the smartphone's role in empowerment, findings suggest that project made important strides in elevating DCs' digital competency to a basic level, with statistically significant improvements across all domains, improving their self-efficacy, and encouraging extra-project phone use. However, the evidence stops short of suggesting that smartphone access played a role in empowering digital champions. Physical barriers to smartphone use, such as limited data, lack of signal, and poorly functioning devices discouraged use as did human and social barriers. Most digital champions have a very narrow view of smartphone capabilities, in part conditioned by the limited scope of the training and the very nascent status of smartphone use within their social networks. Thus, there is a tendency to use it for entertainment and social purposes rather than as a medium for taking actions that could yield tangible positive life impacts.

In conclusion, this study underscores that while citizen science holds promise in GBV prevention, it must be paired with expert oversight, ethical safeguards, and institutional support. Further, the study highlights how injecting smartphones from the outside into an environment will not inevitably usher in a range of positive outcomes. Rather, smartphones are interwoven with a complex set of overlapping systems and structures which condition use. By extension, the findings lend support to those advocating for more restrained enthusiasm regarding the transformative power of smartphones and underscore the need to focus on the transformation, rather than just the technology.

## Data Availability

The datasets generated and/or analyzed during the current study are not publicly available due to the potential risk of participant re-identification, even after anonymization. Reasonable requests for access may be considered on a case-by-case basis and require a data-sharing agreement.
